# The CERN-MEDICIS Isotope Separator Beamline

**DOI:** 10.3389/fmed.2021.689281

**Published:** 2021-09-06

**Authors:** Yisel Martinez Palenzuela, Vincent Barozier, Eric Chevallay, Thomas E. Cocolios, Charlotte Duchemin, Pascal Fernier, Mark Huyse, Laura Lambert, Roberto Lopez, Stefano Marzari, Joao P. Ramos, Thierry Stora, Piet Van Duppen, Alexey Vorozhtsov

**Affiliations:** ^1^KU Leuven, Instituut voor Kern-en Stralingsfysica, Leuven, Belgium; ^2^CERN, Geneva, Switzerland

**Keywords:** MEDICIS, radioisotopes, mass separator, radioactive beams, beamline optics

## Abstract

CERN-MEDICIS is an off-line isotope separator facility for the extraction of radioisotopes from irradiated targets of interest to medical applications. The beamline, between the ion source and the collection chamber, consists of ion extraction and focusing elements, and a dipole magnet mass spectrometer recovered from the LISOL facility in Louvain-la-Neuve. The latter has been modified for compatibility with MEDICIS, including the installation of a window for injecting laser light into the ion source for resonance photo-ionization. Ion beam optics and magnetic field modeling using SIMION and OPERA respectively were performed for the design and characterization of the beamline. The individual components and their optimal configuration in terms of ion beam extraction, mass separation, and ion transport efficiency is described, along with details of the commissioning and initial performance assessment with stable ion beams.

## 1. Introduction

The availability of isotopes for use in the medical field traditionally relies on the extraction of radionuclides from nuclear reactors or targets irradiated by medical cyclotrons or proton/electron accelerators. The radioisotopes that are currently commercially available for nuclear medicine do not necessarily possess the optimal nuclear properties for the treatment or diagnostics of cancer. In this respect, several more promising candidates across the entire nuclear landscape have been identified. Efforts to isolate these and test them for nuclear medicine applications are ongoing.

The ISOLDE (Isotope Separator On Line DEvice) laboratory (1, 2) at CERN has the capability of offering to experimentalists >1,000 isotopes in the form of mass-separated ion beams by using the ISOL (Isotope Separator On-Line) method (3). The diversity and purity of these beams allows for the collection of high-specific activity samples of promising isotopes for the nuclear-medicine community. For example, ^149^Tb (collected from ISOLDE) emits both α and β^+^ particles, enabling targeted therapeutic use and PET imaging to be combined (4). ^152^Tb and ^155^Tb (also produced at ISOLDE) have proven to be suitable candidates for PET and SPECT imaging (5, 6). At ISOLDE, however, the production of radionuclides for medical applications is somewhat restricted by the prioritization of the fundamental nuclear physics research programme. To address this, the CERN-MEDICIS (MEDical Isotopes Collected from ISOLDE) laboratory was constructed to operate parasitically alongside ISOLDE, making use of targets irradiated either at ISOLDE or elsewhere. Both ISOLDE and CERN-MEDICIS are now the main producers of ^155^Tb (7, 8).

CERN-MEDICIS is referred to as an “off-line” isotope separator facility (meaning that it is not directly coupled to a driver beam for isotope production). The production occurs first elsewhere, and only isotope extraction and mass separation take place at MEDICIS. For medical isotope production this is not necessarily disadvantageous since the isotopes of medical interest typically have half-lives of the order of hours to days.

In this paper the MEDICIS beamline elements (ion extraction, focusing and mass separation) are described along with the steps taken to ensure compatibility with standard ISOLDE targets and the space constraints of the MEDICIS bunker. The first results on the production of stable ion beams are also discussed.

## 2. Technical Description of the Beamline Elements

**Figure 1:**
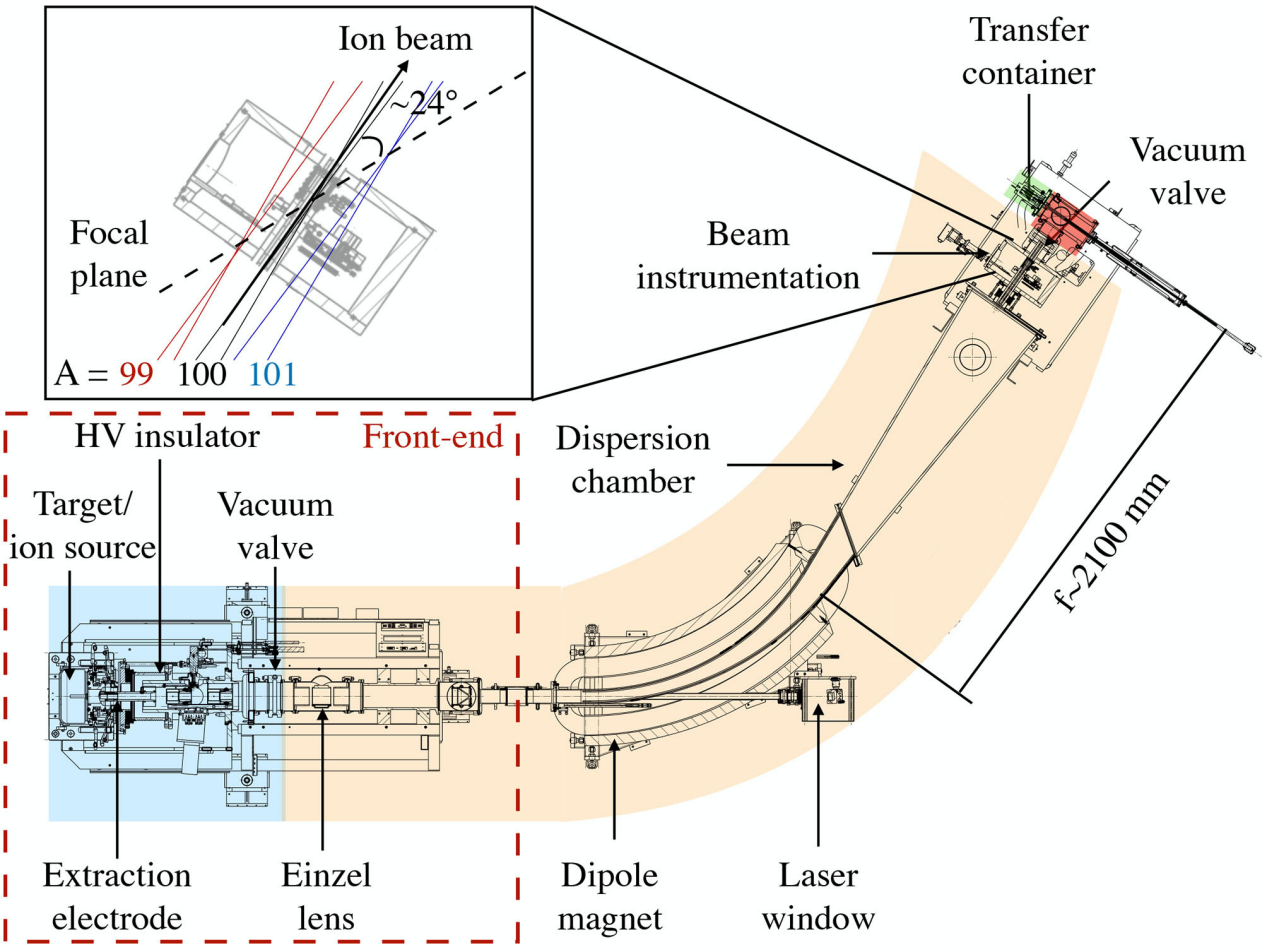
CERN-MEDICIS beamline with the main elements highlighted. The inset shows the inclination of the focal plane with respect to the ion beam in the central trajectory (A/q = 100). This plane is determined by the focal distance of ion beams with mass A/q = 99, 100, and 101. The different pumping sectors—target, separator, collection box, and transfer container—are shown in blue, orange, red, and green, respectively.

**Figure 2:**
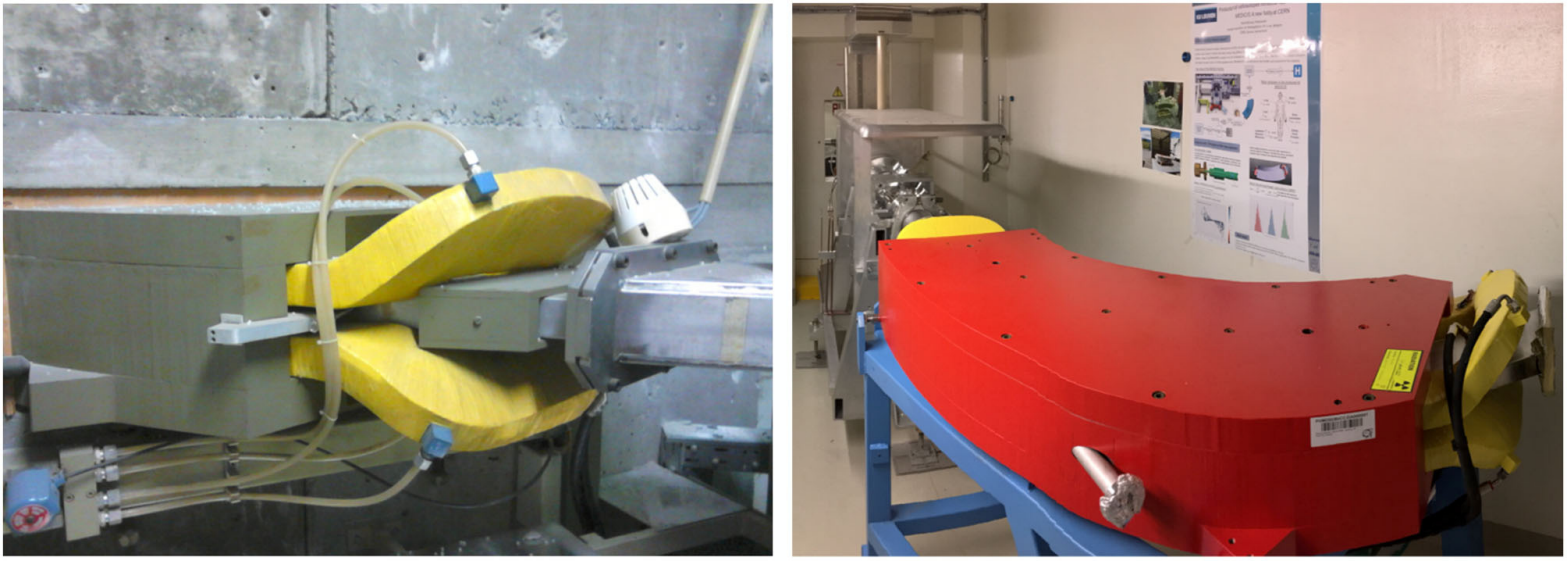
Mass separator donated by KU Leuven to the MEDICIS experiment while at the LISOL facility **(Left)** and once installed at the MEDICIS bunker **(Right)**. The window that was installed to allow lasers to be sent toward the ion source for resonance ionization is clearly seen in the right-hand image.

**Figure 3:**
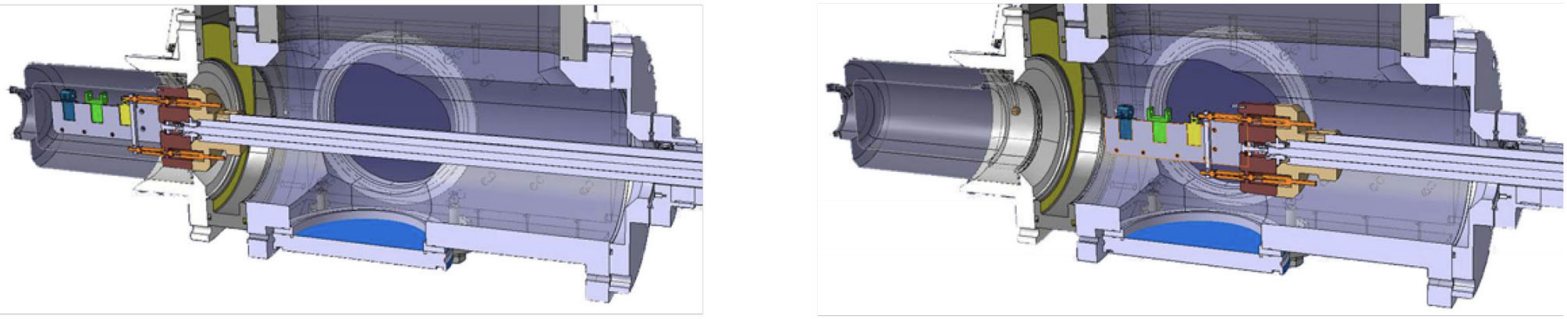
Sample holder positioning. Three different sample positions are shown (blue, green, and yellow). A vacuum valve separates the sample container from the collection box. Once the container is attached to the collection box, the valve is opened, the samples moved into the collection box, and the vacuum valve is closed. Fresh samples to be placed at irradiation position **(Left)** and samples in position to be irradiated, docked in the transport box **(Right)**.

The beamline is located in the so-called MEDICIS bunker. The main elements of the beamline are highlighted in Figure 1 and are described in the list below:

- **Target and ion source unit**: MEDICIS makes use of ISOLDE-standard target/ion source units. The target consists of a tantalum tubular oven of 2 cm diameter and 20 cm length. In a future stage of the development, a larger diameter target container will be used to improve production rates by accounting for the broadened proton beam spot at the ISOLDE irradiation point, located between the primary ISOLDE production target and the proton beam dump. The target container is connected to the ion source via a transfer line. The first beams, both stable and radioactive, were produced at the CERN-MEDICIS facility using a standard ISOLDE surface ionizer. This is a tubular cavity of 3 mm inner diameter and 34 mm length typically made of tantalum, rhenium or tungsten (9, 10). This geometry served as an input for particle tracking, emittance, and mass resolving power calculations using the SIMION software for the characterization and optimization of the beamline elements. SIMION enables the tracking of charged particles when traveling through a region of space in the presence of electromagnetic fields, by solving the equation of motion as a function of their position and velocity (11).
- **Front-end**: A spare ISOLDE front-end 5 (FE5) was adapted for use at MEDICIS taking into account the limited space available in the laboratory. The main components are the electrically isolated target coupling flange usually held at a potential of 30 kV (and up to 60 kV possible), and a ground-potential extraction electrode placed after an acceleration gap of 50–100 mm from the ion source exit. Due to the space constrains in the bunker, an einzel lens was used instead of an electrostatic quadrupole triplet to shape the ion beam downstream of the extraction electrode. It consists of three separate cylindrical electrodes. The radius of the electrodes are 45 mm and their length 77, 90, and 77 mm, respectively. The gap between them is 20 mm. The outer electrodes are at ground, while the central one is kept at a potential in the range of 18 kV (for a beam energy of 30 keV), and is adjusted depending on the focal length required to obtain a parallel beam at the entrance of the magnet. This is a requirement for horizontal and vertical focusing at the focal plane for the kind of magnet employed (12). Between the extraction electrode and the einzel lenses, an X-Y electrostatic deflector is used to adjust for misalignments that may cause the transport of the beam with a wrong angle. This can be achieved by applying a voltage of ±5 kV to the deflectors. The initial position of the vacuum valve in the separator sector was changed to reduce the pumping volume as well as the required volume of the gas storage tanks receiving the radioactive gas load. The final geometry is seen in Figure 1. More details can be found in (9).
- **Beamline vacuum**: The operational vacuum pressure for the different sectors of the MEDICIS beamline are: 10^−5^–10^−6^ mbar for the target sector, 10^−7^ mbar for the separator sector, and 10^−6^ mbar for the collection box and transfer container. The volume of these sectors are ~ 11, 375, 15, and 0.33 L, respectively. The different regions are highlighted in Figure 1. As the pumped volume contains radioactive isotopes, the contaminated gas is stored in tanks located behind the MEDICIS bunker. The gas is held there until the radioactivity falls to a sufficiently low level to allow release into the atmosphere. For more details on the MEDICIS vacuum system [see (13)].
- **Mass separator**: The mass separator dipole magnet used at CERN-MEDICIS was provided by the University of Leuven (KU Leuven) in Belgium, and is the magnet from the former Leuven ISOL (LISOL) facility (12). It is a 55° double focusing magnet with a bending radius of 1.5 m. The entrance angle of the magnet is 90 and 35.5° at the exit. The magnet has a curved, H-type yoke made of solid iron, with an aperture of 200 mm width and 55 mm height (full mechanical aperture). It has an indirect water cooled, bedstead coil electrically connected in series. The required maximum integrated field strength is provided by 90 turns per coil with a current of 120 A.
  Ions are bent in the horizontal plane following a 55° curvature radius. The integrated field homogeneity ΔB_*y*_d_*z*_/B_*y*_(0,0,z)d_*z*_ must be better than 2·10^−4^ inside the rectangular homogeneous field region, where *z* is the distance along the central trajectory.
  The mass separator was modified to enable use with a resonance ionization laser ion source (MELISSA—MEDICIS laser ion source setup) (14). The laser ion source is crucial when surface or electron-impact ionization is either not sufficiently efficient or selective. A window for the lasers with a 33 mm diameter was incorporated to the vacuum chamber of the magnet to allow a clear line-of sight to the ion source [see Figure 2 (Right)]. For this, a hole in the magnet yoke had to be machined. A shutter was installed in front of the window to prevent ion beam implantation when, for example, the dipole magnet is off but the high voltage is on, which may cause a reduction in transparency.
- **Beam diagnostics instrumentation**: The beam instrumentation is located inside the collection box. Faraday cups are used, one for total beam current measurements (before the mass separator) and one to measure the separated beam (in the collection box). Wire scanners are used for beam profile and quality assessment in the transversal plane. The focal plane distance and inclination with respect to the central beam trajectory was determined using the simulation software SIMION. For this, particles with *A*/*q* = 99, 100, and 101 were launched from either side of the central trajectory, upstream of the dipole magnet at 30 keV.
  The angle of the focal plane with respect to the central trajectory is ~24° and the focal plane distance is ~2,100 mm [which agrees with the theoretically calculated distance following matrix formulation (9)] (see inset in Figure 1). These results were used to determine beam instrumentation specifications. The scanner moves along the focal plane with the calculated inclination angle. The collection point is then placed as close as possible to the focal point for the beam on the central trajectory.
- **Sample collection system**: This is also located inside the collection box. Up to three samples can be placed in the transfer container separated by 15 mm with a size of 10 × 10 mm. In front of the holder for the sample plates, a collimator and electron deflector are placed. The current in the sample should be maximized while it should be minimized in the collimator. The position of the sample plate is controlled by an arm that moves perpendicularly to the beam and intercepts the beam in the central trajectory as shown in Figure 3.

## 3. Beamline Optimization and Characterization

### 3.1. Mass Resolving Power

The mass resolving power (MRP) depends on the properties of the magnet and on the optical properties of the ion beam. Here we will use the MRP definition given in (12):

(1) $$\begin{array}{l} MRP=d\cdot \frac{M}{FWHM} \end{array}$$

where *d* is the distance between two adjacent peaks with masses *M* and *M*+1 and FWHM (with units of distance) is the full width at half maximum of a beam of ions with mass *M* in the focal plane of the separator.

The MRP was calculated as a function of the einzel lens voltage for the original configuration (geometry 1), in which the vacuum valve for vacuum sector 1 (target sector) is placed downstream of the einzel lens. The results are compared with a new geometry (geometry 2), in which the vacuum valve is instead placed right after the target. This way, the target sector is isolated from the rest of the beamline (see Figure 1) reducing its pumping volume and thereby reducing the required volume of the contaminated gas storage tanks. Other elements of the beamline were also discarded/replaced during the modifications. The einzel lens was moved 80 mm away from the ion source to accommodate this modification. More details can be found in (15).

**Figure 4:**
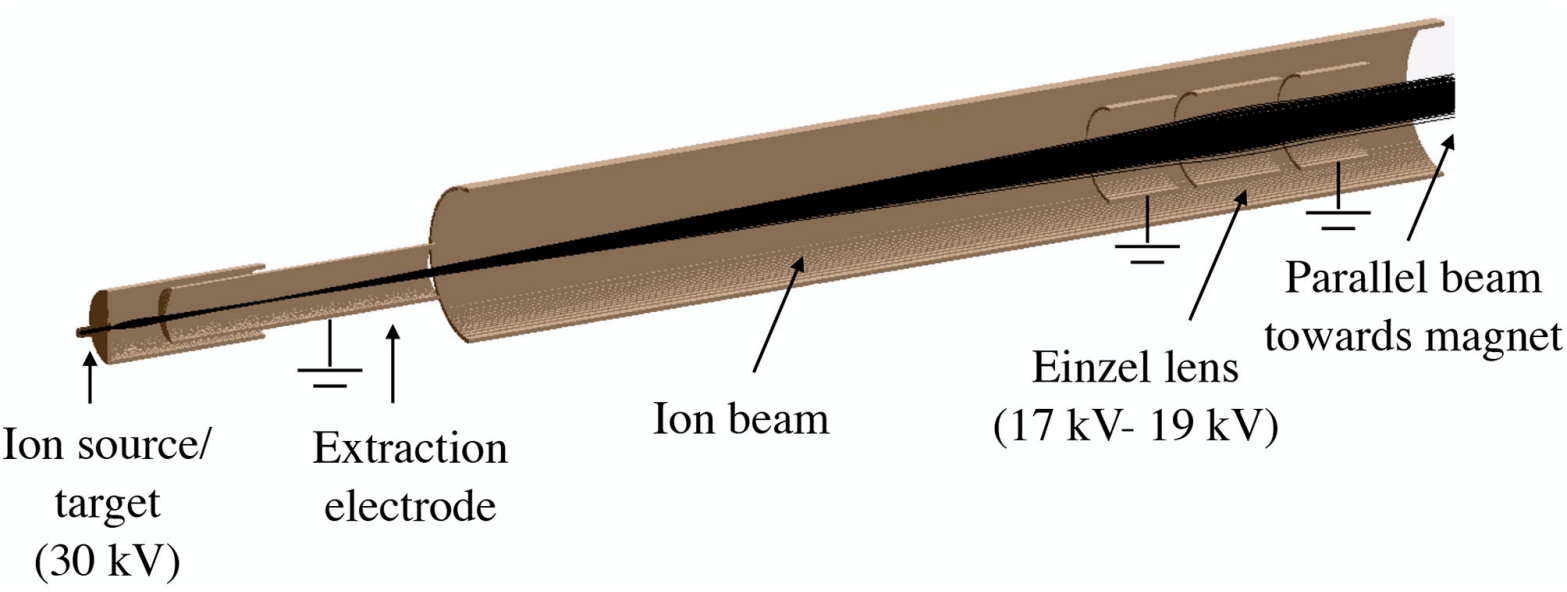
Geometry of the MEDICIS beamline simulated with SIMION showing the electromagnetic elements included in the simulations. The black lines show the charged particle trajectories.

SIMION was used to assess the effect of such modification on the MRP. Figure 4 shows the main elements taken into account to perform the simulations.

Ions of *A*/*q* = 99, 100, and 101 were launched from a surface ion source as shown in Figure 4, with an homogeneous distribution inside the ion source that follows a Gaussian energy distribution [with mean energy of 0.25 eV (~3,000 K) and a 10% energy spread]. Ions drift toward the exit of the hot cavity due to a longitudinal voltage drop of ~2 V (this voltage gradient was also taken into account for the simulations) and are extracted due to the field penetration created by the extraction electrode. The ions are then accelerated toward the dipole magnet and focused at the focal plane.

**Figure 5:**
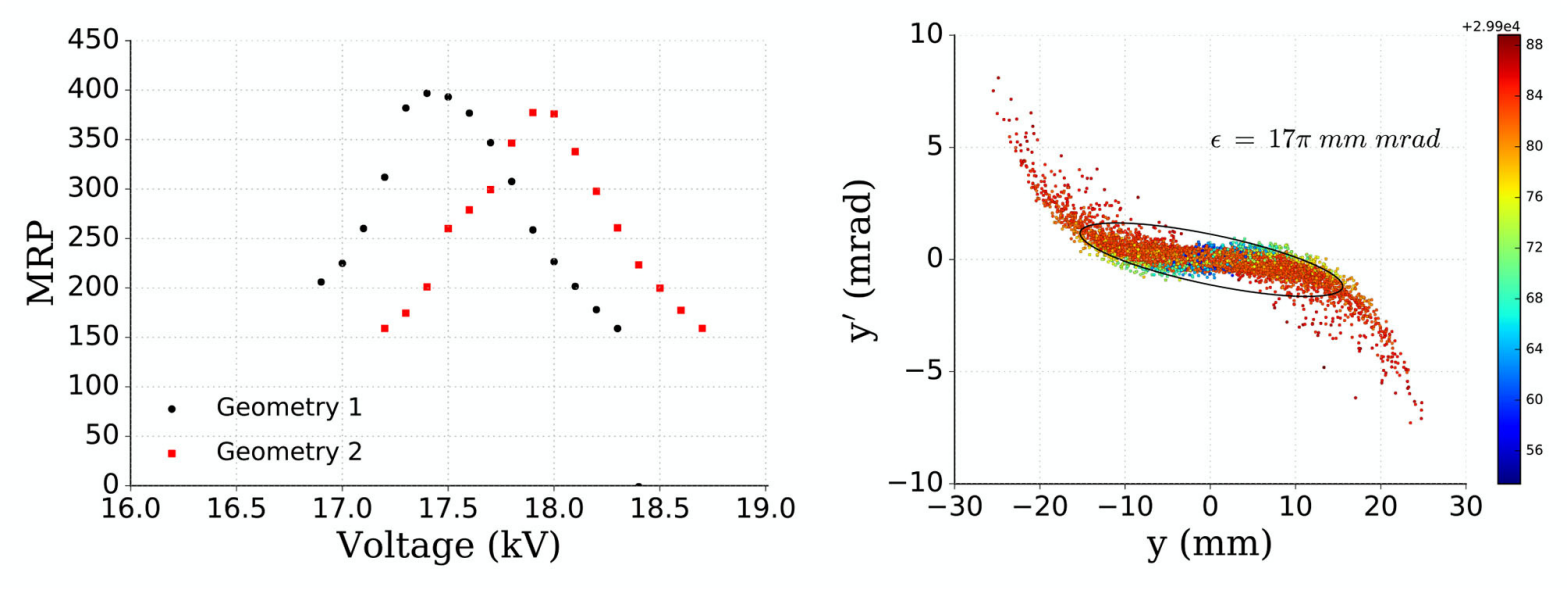
MRP vs. einzel lens voltage for geometry 1 (black circles) and geometry 2 (red squares). These geometries differ in the position of the einzel lens within the beamline, geometry 2 having the einzel lens further away from the ion source **(Left)**. Beam emittance for geometry 2 (final geometry) with the ellipse encompassing 95% of the simulated events. The colorbar is the energy range of the particles: 2.99e4 + 0.0056 keV to 0.0088 keV **(Right)**.

The results of the simulations are shown in Figure 5 (Left). It can be clearly seen how the voltage of the einzel lens needs to be adjusted to maximize the MRP, which is also influenced by such displacement dropping from 400 to 380. For an ion beam with A/q = 100, the MRP is approximately halved with only a ~3% deviation of the optimal einzel lens voltage. This MRP sensitivity to the strength of the einzel lens or its positioning is due to the requirement of a parallel beam entering the separator magnet.

The simulated beam emittance was computed at the entrance of the magnet and it is displayed in Figure 5 (Right). The ellipse that has been drawn and the values computed encompass 95% of the simulated events.

### 3.2. Incorporation of the Laser Window

The installation of the laser window [Figure 2 (Right)] required a perforation of the magnet yoke. To make sure this modification wouldn't affect the magnetic field homogeneity, magnetic field calculations of the dipole magnet using the Opera-3D/TOSCA program were performed. OPERA is a software suite for electromagnetic, thermal and structural simulations (16).

**Figure 6:**
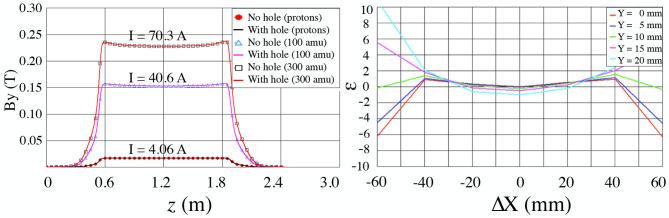
Comparison between OPERA models without and with the drilled hole necessary for the laser beam access (no hole—symbols/with hole—solid lines) for three different values of the current I (A), needed to have masses A/q = 1, 100, and 300 in the central trajectory, respectively **(Left)**. Relative integrated field error [10^−4^] for I = 70.3 which corresponds to A = 300 amu at 30 keV for a magnet with the laser window incorporated **(Right)**.

The magnetic field distribution along the central trajectory for three different values of the current was determined. Figure 6 (Left) compares the magnetic field along the magnet central trajectory for the original structure of the magnet (no hole) and when the hole in the yoke (40 mm outer diameter for the laser window) is taken into account.

It is seen then that the impact of the hole in the return yoke (laser window, with hole) on the field strength is negligible compared to model without the hole.

Calculations were performed to determine the homogeneous field region of the magnetic field. The field was calculated at different heights [Y-direction, different colors as shown in Figure 6 (Right)]. The X-axis represents the shift from the central trajectory.

From this plot we can conclude that in order to keep an integrated field homogeneity better than ±2·10^−4^, the beam should stay within a rectangle of 40 mm in the X-direction (horizontal) and 20 mm in the Y-direction (vertical).

## 4. First Mass Separated Ion Beams at MEDICIS

The production of the first stable (non-radioactive) beams at MEDICIS was performed with a standard ISOLDE surface ion source (17) as well as the ISOLDE variant of the FEBIAD (18), known as the VADIS (Versatile Arc Discharge Ion Source) (19). The correct functioning of the following services were verified: target coupling, target and ion source heating and cooling, high voltage, einzel lens, and deflector voltage, magnet cooling as well as proper operation of the scanners and Faraday cups. This has been demonstrated by the observation of mass-separated ion beams of Na, K, Al, La (surface ion source) and the noble gases He, Ne, Kr, Ar, Xe (VADIS).

**Figure 7:**
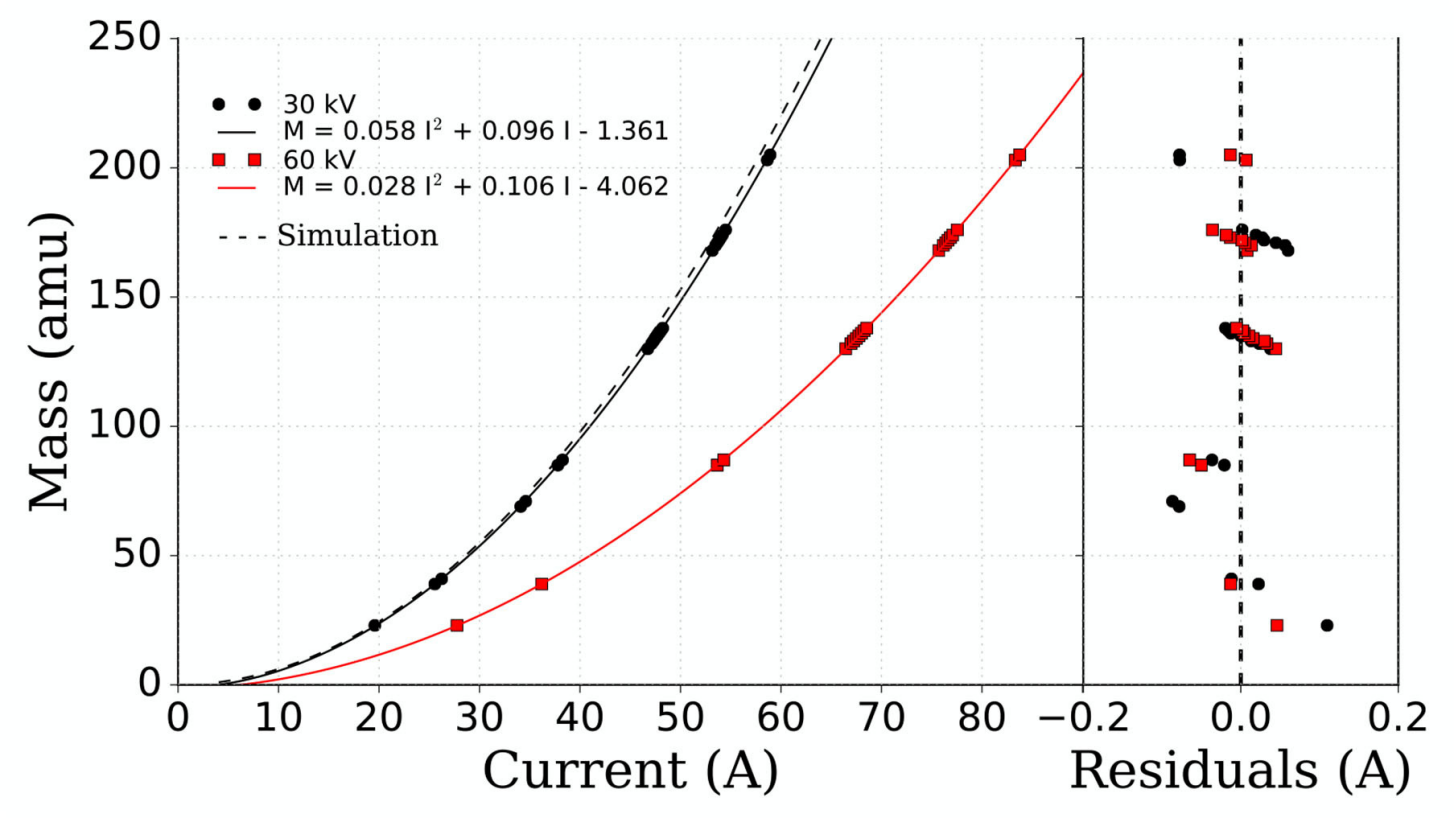
Calibration curve of the MEDICIS dipole magnet for 30 and 60 kV extraction voltage. The solid lines are second order polynomial fits to the experimental data. The simulated curve obtained from OPERA calculations is represented by the dashed line. The fit residuals are shown in the right hand side of the figure.

Figure 7 shows a magnet calibration using the most intense beams produced by the surface ion source. The simulated curve for a 30 kV extraction voltage (dashed line), was generated using the technical specifications of the magnet from the manufacturer as described in section 2, by means of OPERA simulations and electromagnetism calculations. The experimental points (and 2nd order polynomial fitted curve shown in black) lie closely on this line, and the similarity of these curves serve as a validation of the OPERA simulations. The magnet calibration for 60 kV extraction voltage is also represented by the red curves.

**Figure 8:**
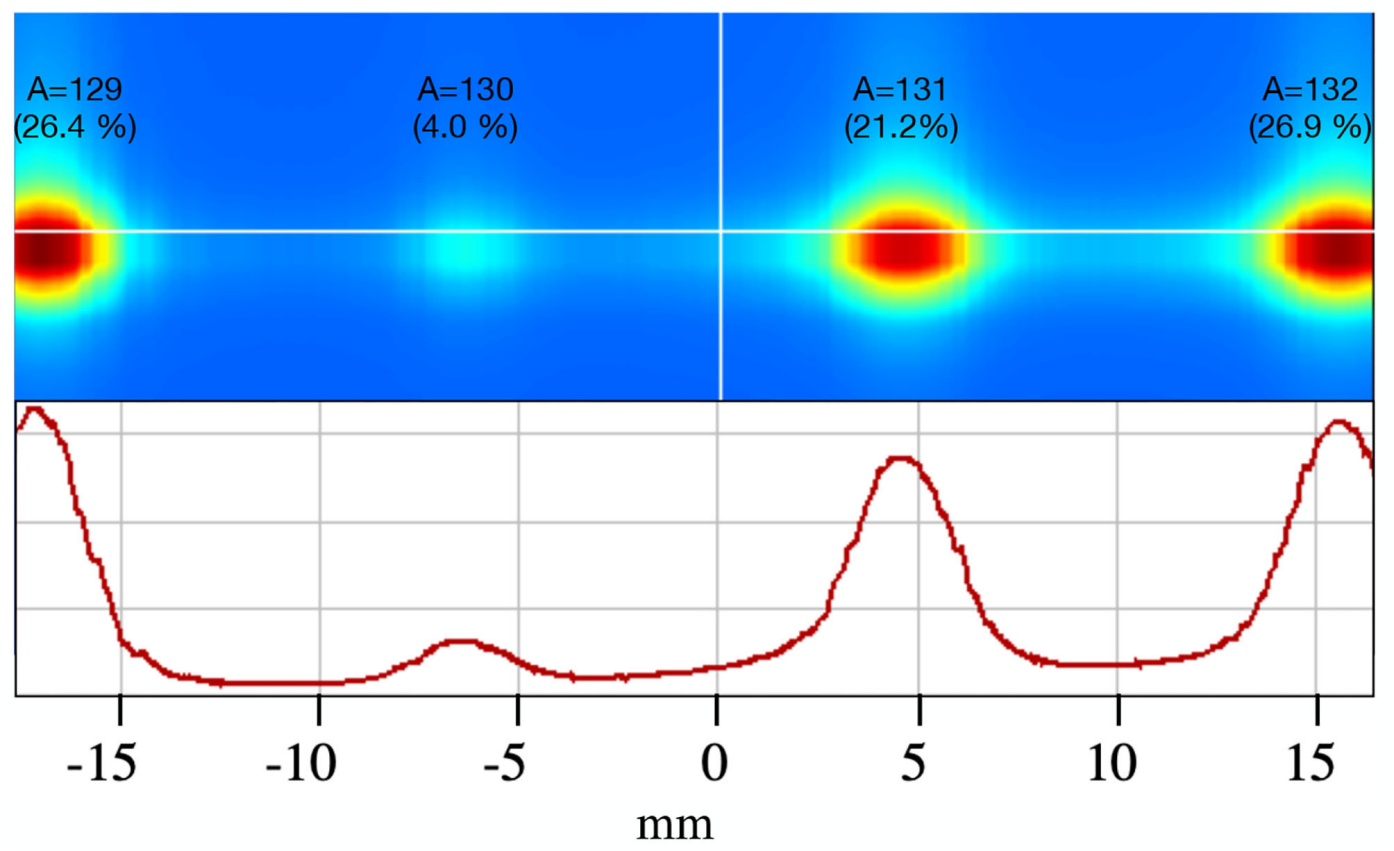
Xe beam profile with A = 129, 130, 131, and 132 and respective natural abundances. The relative beam intensities and beam spotsize are represented via a heatmap **(top)** with red and blue being the maximum and minimum values, respectively. 2D plot of the Xe beam profile **(bottom)**.

A gas mixture of helium, neon, argon, krypton and xenon (20% each) was injected into the target/ion source unit (a VADIS in this case) using a gas leak. Figure 8 shows the beam profile in the xenon region with masses 129, 130, 131, and 132 visible and with the expected natural abundances. The extraction electrode was 40 mm away from the exit of the ion source. The measured total beam was 1.33 μA and the separated beam for ^132^Xe equalled 135 nA. The cathode temperature was kept at 1,950°C. The beam transmission, calculated by comparing the sum of the mass separated beams to total beam before separation was determined to be ~90%.

The mass resolving power (MRP) was calculated using Equation 1, based on information extracted from Figure 8. Considering FWHM~3 mm for A = 131 and *d*_(131−132)_~12 mm, a value for the MRP = 500 is obtained.

## 5. Conclusions

A dipole magnet mass separator, previously used at the LISOL experiment for over 40 years, was recovered and installed in a compact bunker in the MEDICIS laboratory for the extraction of radioisotopes of medical interest. The magnet was modified for compatibility with resonance ionization by installing a laser window. It was demonstrated by means of OPERA simulations that the field homogeneity was not affected by such modification.

SIMION was used to show that the mass resolving power depends on the divergence of the beam entering the magnet, which can be optimized by adjusting the einzel lens voltage. It was also shown that the required voltage for maximum mass resolving power depends on the longitudinal position of the einzel lens with respect to the extraction. The dependence of the mass resolving power on the einzel lens voltage and longitudinal position highlights the importance of these parameters when optimizing the MEDICIS beamline. This is crucial if there are more intense neighboring components in the mass spectrum since the use of a Faraday cup alone may give an ambiguous result during beam tuning optimization.

The first stable beams produced during the commissioning phase showed that the facility is operating in line with expectations and indicated its suitability for full scale operation.

## Data Availability Statement

The original contributions presented in the study are included in the article/supplementary material, further inquiries can be directed to the corresponding author/s.

## Author Contributions

YM conceived the paper, prepared the figures and plots, took part in the beamline and first beams commissioning, performed the SIMION simulations. VB and SM provided the technical drawings for simulations. RL and AV performed the OPERA simulations. EC, CD, PF, and LL provided data regarding the magnet calibration. TC, MH, TS, PV, and JR supervision and assistance with the manuscript. All authors contributed to the revision of the manuscript.

## Conflict of Interest

The authors declare that the research was conducted in the absence of any commercial or financial relationships that could be construed as a potential conflict of interest.

## Publisher's Note

All claims expressed in this article are solely those of the authors and do not necessarily represent those of their affiliated organizations, or those of the publisher, the editors and the reviewers. Any product that may be evaluated in this article, or claim that may be made by its manufacturer, is not guaranteed or endorsed by the publisher.
